# Determinants of knowledge and positive perception of HIV pre-exposure prophylaxis: the role of age and sexual orientation in patients of specialized STI and transgender health services

**DOI:** 10.1186/s12889-025-25812-4

**Published:** 2025-12-02

**Authors:** Paula Knoch Mendonça Gil, Danilo dos Santos Conrado, Ana Isabel do Nascimento, João Cesar Pereira da Cunha, Gabriel Serrano Ramires Koch, Camila Guadeluppe Maciel, Cláudia Du Bocage Santos-Pinto, Everton Falcão de Oliveira

**Affiliations:** 1 Programa de Pós-Graduação em Doenças Infecciosas e Parasitárias, Universidade Federal de Mato Grosso do Sul, Campo Grande, MS Brazil; 2 Secretaria Municipal de Saúde Pública, Campo Grande, Mato Grosso do Sul Brazil; 3 Faculdade de Medicina, Universidade Federal de Mato Grosso do Sul, Campo Grande, MS Brazil

**Keywords:** Pre-Exposure prophylaxis, Knowledge, Barriers to access to health care, Health risk behaviors, Epidemiology, Disease prevention

## Abstract

Pre-exposure prophylaxis (PrEP) is one of strategies for preventing human immunodeficiency virus infection. However, limited knowledge and perception of PrEP may impact the access to this prophylaxis. This study aimed to assess knowledge and perceptions of PrEP and to identify determinants, both sociodemographic and structural, associated with them among users of specialized public health services. This is a cross-sectional study based on data obtained through interviews conducted with patients who sought specialized services in STI on a spontaneous basis, as well as with routine patients at an outpatient clinic for transgender healthcare. A total of 142 users of the Brazilian Unified Health System services were interviewed. Eligible participants were individuals aged ≥ 18 years who sought care at the recruitment sites, were not currently using PrEP, and self-reported no prior HIV diagnosis. Data collection at these two locations took place between October 2021 and April 2022. Most participants were young, heterosexual, non-white men with higher education. Condom use was often inconsistent. Limited PrEP knowledge emerged as a major gap, while younger users showed a more favorable perception of PrEP. Understanding sociodemographic determinants of PrEP knowledge and perception highlights critical barriers and facilitators to its broader implementation. These findings may inform targeted strategies to enhance PrEP awareness, uptake, and adherence within Brazil’s public health system.

## Introduction

 Limited knowledge about HIV prevention methods, particularly among key populations at higher risk of infection, can constitute a significant barrier to accessing and adopting available methods like pre-exposure prophylaxis (PrEP) and other combination prevention strategies [1, 2].

The first randomized clinical trials carried out on the effectiveness of PrEP were published in the 2000 s [3–5] and, even after more than a decade of successful findings showing high protection in preventing HIV and the safety of PrEP, the knowledge dissemination on this topic still represents a challenge in the context of public health [6].

In Brazil, PrEP has been available free of charge through the Unified Health System since 2018 [7]. However, its distribution in some Brazilian cities is concentrated in specialized centers such as Testing and Counseling Centers, Specialized Care Services, and STI/AIDS reference and training centers. This concentration can pose a structural barrier to initiating and adhering to PrEP [8].

The first Brazilian guidelines for PrEP recommendation and provision prioritized key populations, such as gays and men who have sex with men, sex workers, transgender people, and HIV serodiscordant couples, as these groups have been historically more affected by the HIV/AIDS epidemic [7]. In 2022, the Ministry of Health updated the PrEP eligibility criteria, expanding access to individuals aged 15 and older, weighing 35 kg or more, who are sexually active and face higher-risk contexts for HIV acquisition. PrEP is now recommended for non-HIV-infected individuals who meet prophylactic criteria based on their sexual behaviors, number of sexual partners, inconsistent condom use, and other associated risk factors. The new guidelines encourage interested and motivated individuals to use PrEP medication in accordance with these updated criteria [9].

Despite recent advances, barriers to accessing and expanding PrEP remain, including high discontinuation rates, stigma, and broader social and structural determinants such as economic constraints and limited knowledge [6, 8, 10–12]. This study was motivated by the need to explore barriers and facilitators of PrEP access and adherence, extending beyond those already documented in the literature and considering the influence of public policies and the broader public health impact of PrEP among vulnerable populations. Evidence remains scarce on PrEP use within the Brazilian Unified Health System, particularly in medium-sized cities and across diverse service contexts such as specialized STI clinics and transgender healthcare services. Given PrEP’s essential role in HIV prevention and its potential to influence population-level health outcomes, limited awareness or misconceptions may contribute to poor adherence and a higher incidence of STIs. Understanding both individual and structural determinants of PrEP knowledge and perception is essential to inform equitable access and improve the implementation of PrEP programs. In this context, this study aimed to assess knowledge and perceptions regarding PrEP and to identify determinants (both sociodemographic and structural, such as service organization and accessibility) associated with them among patients attending specialized STI and transgender healthcare services in Campo Grande, Mato Grosso do Sul, Brazil.

## Methods

### Study design and population

This cross-sectional study was based on interviews with patients seeking care at the Testing and Counseling Center (*Centro de Testagem e Aconselhamento*, CTA), a specialized STI counseling and testing facility operating on a walk-in basis, as well as with routine patients at the Multidisciplinary Outpatient Clinic for the Care of Transgender Individuals (Transgender Health Clinic), which provides services by appointment. The CTA, located in central Campo Grande, is the city’s only PrEP provider, while the Transgender Health Clinic is situated nearby. Both sites are public healthcare facilities within the Brazilian Unified Health System, offering free, universal access to care. Data collection at these locations occurred between October 2021 and April 2022.

The sample size was defined by feasibility. Given the characteristics of the study setting and data collection during the COVID-19 pandemic, we used consecutive sampling and included all eligible individuals who attended the services on the data collection days. No a priori sample size or power calculation was performed.

All individuals who sought care at either of the two recruitment sites on the scheduled data collection days were considered for inclusion. Exclusion criteria were: being younger than 18 years old, currently using PrEP, or self-reporting a previous HIV diagnosis at the time of invitation. Data were collected through face-to-face interviews conducted by trained members of the research team using a structured questionnaire. All interviews were conducted individually in a private room within the health facility to ensure confidentiality and participant comfort.

### Data collection instrument and measurements

The data collection instrument was developed by the research team and guided by a theoretical framework addressing the implementation of strategies in health services [13]. This framework outlines four categories of characteristics, each comprising determinants that may act as barriers or facilitators depending on the study context. Based on this foundation, the questionnaire was organized into three sections: (1) sociodemographic and sexual health–related variables, including elements relevant to PrEP eligibility; (2) knowledge about PrEP; and (3) perceptions regarding PrEP.

#### Sociodemographic and behavioral variables

The first section included information on age (years), number of steady sexual partners in the last 6 months and in the last 30 days, gender, and sexual orientation (heterosexual; LGBTQIA+). Participants were also asked about self-reported skin color (White; Black; Brown/mixed race; Asian descent; Indigenous), religion (Catholic; Evangelical; Spiritist; Umbanda, Candomblé, or other Afro-Brazilian religions; other; without religion; atheist), educational level (primary: 1–9 years of formal education; secondary: 10–12 years of education; higher education: partial or complete university-level education), and monthly income measured in Brazilian minimum wages (< 1; 1–5; ≥5). At the time of data collection, one Brazilian minimum wage was BRL 1,100 (approximately USD 202, based on an exchange rate of 1 USD = 5.44 BRL). Sexual behavior variables included sexual activity in the past 6 months (yes/no), steady partnership in the past 6 months (yes/no), condom use during sexual intercourse (always; sometimes; never), condomless sex in the past 6 months (yes/no), and condomless sex with a person living with HIV/AIDS in the past 6 months (yes/no; don’t know).

#### Knowledge about PrEP

The second section assessed knowledge using 18 true/false items. This instrument was adapted from Emmanuel et al. [14] and expanded by our group, which previously applied it to assess PrEP knowledge among healthcare professionals in the same settings [15]. In the absence of a standardized threshold in the literature for knowledge instruments of this type, we adopted a cutoff of 60% correct responses (≥ 11 of 18 correct answers), consistent with our previous work. This pragmatic criterion, commonly used in educational contexts to distinguish adequate from inadequate performance, was considered appropriate for identifying subgroups with insufficient knowledge requiring targeted interventions. Unanswered items were classified as incorrect.

#### Perceptions about PrEP

The third section comprised a single multiple-choice question addressing perceptions of PrEP, including positive, negative, and neutral statements. For analysis, responses were categorized as either *positive* or *negative/indifferent*. Participants who selected both a positive option and one or more negative/indifferent options were categorized as *negative/indifferent*.

#### Validation and data collection procedures

To ensure content validity, six experts with doctoral degrees in STIs or Primary Healthcare were invited to review the interview guide, of whom four participated. Their evaluation focused on clarity, relevance, and comprehensiveness, and all suggested modifications were incorporated into the final instrument. Prior to data collection, the instrument was further reviewed by two independent infectious disease physicians (both with PhDs in Medicine/Infectious Diseases) with extensive experience in HIV/AIDS and PrEP care, to assess reliability and coverage of key knowledge domains.

Data collection was carried out over seven months, with researchers present at each recruitment site for two four-hour sessions per week. Days and time slots were selected in consultation with staff to coincide with periods of higher patient flow. Interviews were conducted and responses recorded directly in the REDCap platform [16, 17].

### Data analysis

Descriptive statistical analysis was employed to characterize the study population, utilizing frequency distributions of study variables, as well as calculating means, standard deviations, and proportions where applicable.

Statistical association between knowledge about PrEP and variables related to the study population profile were assessed using the chi-square test or Fisher’s exact test, when the assumption of the chi-square test was not met (i.e., when any expected frequency was less than 5). Mean comparison tests were used to verify the statistical association between quantitative variables. The same procedures were used to evaluate the association between the perception and study population profile.

Binomial logistic regression models were used to evaluate the association between the covariates (sociodemographic and behavioral variables) and the study outcomes (knowledge and perception). Variable selection for the multivariable models was conducted in two steps. First, covariates showing an association with a p-value < 0.20 in bivariate analysis were considered for inclusion, following conventional criteria commonly applied in epidemiological studies to avoid excluding potential predictors prematurely. Second, a stepwise algorithm (both backward and forward) using the Akaike Information Criterion (AIC) was applied to select the most relevant variables, control for potential confounding, and define the best-fitting model. Multicollinearity was assessed using the variance inflation factor (VIF). The Hosmer-Lemeshow test and the le Cessie-van Houwelingen test were used as goodness-of-fit measures for the model. The significance level of 5% (α = 0.05) was adopted for all hypothesis tests performed.

All statistical analyses were performed using R software (version 4.3.2).

### Ethical aspects

The study was approved by the UFMS Research Ethics Committee (CAAE 39688520.0.0000.0021; Protocol Number 4.477.496), and procedures were in accordance with the ethical standards of this committee and with the Helsinki Declaration of 1975, as revised in 1983.Written informed consent was obtained from all participants.

## Results

At total, 142 participants were interviewed, and of these, 100 (70.4%) in the specialized services in STI and 42 (29.6%) in the outpatient clinic specialized in transgender population health. The average age of users was 31 years (SD = 10.2 years) and the most frequent age group was 18 to 29 years (53.5%). Participants of non-white race/color (67.6%), cisgender (73.9%), heterosexual (63.4%), Catholic religion (27.5%), with higher education (49.3%) and with a monthly income consistent with middle-class standards in Brazil were the most prevalent (Table 1).

**Table 1:** Characterization of the study population according to knowledge and perception about PrEP (*n*= 142)

	Total	Knowledge		*p*-value	Perception		*p*-value
		Less than 60%	60% or more		Positive	Negative/ indifferent	
Age (years)							
Mean (SD)	31.0 (10.2)	31.4 (10.4)	29.5 (9.6)	0.334	29.5 (9.0)	33.5 (11.8)	0.026
Number of fixed sexual partnerships in the last 6 months							
Mean (SD)	3.3 (9.3)	1.8 (1.4)	8.9 (20.1)	0.001	2.5 (3.7)	4.7 (15.2)	0.225
Number of fixed sexual partnerships in the last 30 days							
Mean (SD)	1.1 (1.1)	1.0 (0.7)	1.6 (1.9)	0.016	1.1 (0.8)	1.1 (1.4)	0.927
Gender	***n*** **(%)**	***n*** **(%)**	***n*** **(%)**	***n*** **(%)**	***n*** **(%)**	***n*** **(%)**	
Cisgender	105 (73.9)	82 (75.9)	26 (24.1)	1.000	67 (62.0)	41 (38.0)	0.698
Transgender	34 (24.0)	26 (76.5)	8 (23.5)		23 (67.6)	11 (32.4)	
Others	3 (2.1)	-	-		-	-	
Sexual orientation							
Heterosexual	90 (63.4)	79 (87.8)	11 (12.2)	< 0.001	57 (63.3)	33 (36.7)	1.000
LGBTQIA+	52 (36.6)	29 (55.8)	23 (44.2)		33 (63.5)	19 (36.5)	
Skin color							
White	46 (32.4)	33 (71.7)	13 (28.3)	0.532	26 (56.5)	20 (43.5)	0.323
Black/Brown/mixed race/Asian descent/Indigenous	96 (67.6)	75 (78.1)	21 (21.9)		64 (66.7)	32 (33.2)	
Religion							
Catholic	39 (27.5)	33 (84.6)	6 (15.4)	0.405	21 (53.8)	18 (46.2)	0.010
Evangelical	22 (15.5)	18 (81.8)	4 (18.2)		20 (90.9)	2 (9.1)	
Spiritist	10 (7.0)	8 (80.0)	2 (20.0)		3 (30.0	7 (70.0)	
Umbanda, Candomblé or other Afro-Brazilian religions	10 (7.0)	6 (60.0)	4 (40.0)		7 (70.0)	3 (30.0)	
Other	17 (12.0)	11 (64.7)	6 (35.3)		12 (70.6)	5 (29.4)	
Without religion	41 (28.9)	31 (75.6)	10 (24.4)		24 (58.5)	17 (41.5)	
Atheist	2 (1.3)	1 (50.0)	1 (50.0)		2 (100.0)	0 (0.0)	
Missing	1 (0.7)	0 (0.0)	1 (100.0)		1 (100.0)	0 (0.0)	
Educational level							
Primary Education	18 (12.7)	15 (83.3)	3 (16.7)	0.148	9 (50.0)	9 (50.0)	0.212
Secondary Education	54 (38.0)	45 (83.3)	9 (16.7)		32 (59.3)	22 (40.7)	
Higher education	70 (49.3)	48 (68.6)	22 (31.4)		49 (70.0)	21 (30.0)	
Monthly income (in Brazilian minimum wage)							
Less than 1	49 (34.5)	38 (77.6)	11 (22.4)	0.933	34 (69.4)	15 (30.6)	0.583
1 to 5	86 (60.6)	65 (75.6)	21 (24.4)		52 (60.5)	34 (39.5)	
5 or more	5 (3.5)	4 (80.0)	1 (20.0)		3 (60.0)	2 (40.0)	
Missing	2 (1.4)	1 (50.0)	1 (50.0)		1 (50.0)	1 (50.0)	
Sexual relation in the last 6 months							
Yes	126 (88.7)	98 (77.8)	28 (22.2)	0.214	79 (62.7)	47 (37.3)	0.843
No	16 (11.3)	10 (62.5)	6 (37.5)		11 (68.8)	5 (31.2)	
Fixed sexual partnership in the last 6 months							
Yes	81 (57.0)	69 (85.2)	12 (14.8)	0.011	50 (61.7)	31 (38.3)	0.788
No	44 (31.0)	28 (63.6)	16 (36.4)		29 (65.9)	15 (34.1)	
Missing	17 (12.0)	11 (64.7)	6 (35.3)		11 (64.7)	6 (35.3)	
Use of condoms during sexual relations							
Always	33 (23.2)	19 (57.6)	14 (42.4)	0.001	19 (57.6)	14 (42.4)	0.562
Sometimes	55 (38.7)	39 (70.9)	16 (29.1)		34 (61.8)	21 (38.2)	
Never	54 (38.0)	50 (92.6)	4 (7.4)		37 (68.5)	17 (31.5)	
Sexual relation without a condom in the last six months							
Yes	28 (19.7)	19 (67.9)	9 (32.1)	0.322	18 (64.3)	10 (35.7)	1.000
No	114 (80.3)	89 (78.1)	25 (21.9)		72 (63.2)	42 (36.8)	
Sexual relation without a condom with a PLWHA in the last six months							
Yes	5 (3.5)	2 (40.0)	3 (60.0)	0.035	2 (40.0)	3 (30.0)	0.521
No	122 (85.9)	97 (79.5)	25 (20.5)		78 (63.9)	44 (36.1)	
I don’t know	15 (10.6)	9 (60.0)	6 (40.0)		10 (66.7)	5 (33.3)	
PrEP perception							
Positive	90 (63.4)	64 (71.1)	26 (28.9) 8 (15.4)	0.107	-		-
Negative/indifferent	52 (36.6)	44 (84.6)			-		
Percentage of correct answers to questions on PrEP							
Less than 60%	108 (76.1)	-	-	-	64 (59.3)	44 (40.7)	0.107
60% or more	34 (23.9)	-			26 (76.5)	8 (23.5)	

The category 'Missing' includes responses that were not provided by the participants

*Abbreviations*: *PLWHA* People living with HIV/aids, *PrEP* Pre-exposure prophylaxis, *LGBTQIA+* Lesbian, Gay, Bisexual, Transgender, Queer (or Questioning), Intersex, Asexual, and others (the plus sign represents additional identities not explicitly included in the acronym)

The majority (57%) reported having a steady sexual partnership in the last six months and 38% denied using condoms during sexual relations. The frequency of reporting unprotected sex with more than one person in the last 6 months or with people living with HIV/AIDS was 19.7 and 3.5%, respectively (Table 1).

In the univariate analysis, the percentage of correct answers on knowledge about PrEP showed significant associations with several variables: sexual orientation, having steady sexual partnerships in the last six months, average number of steady sexual partnerships in the last six months and in the past 30 days, condom use during sexual intercourse, and engaging in sex without a condom with people living with HIV/AIDS (PLWHA) (Table 1).

A positive perception regarding PrEP was reported by more than half of the participants (63.4%; 90/142). Negative or indifferent perceptions accounted for 20.4% (29/142) and 16.2% (23/142), respectively. Perception was found to be significantly associated with the variables of age and religion (Table 1).

The highest percentage of correct answers regarding PrEP knowledge was for the statements ‘Individuals using PrEP should be tested regularly for HIV and other STIs’ and ‘PrEP is highly effective when used correctly’. In contrast, the lowest percentage was for the statement ‘The use of PrEP is contraindicated during pregnancy.’ However, the percentage of correct answers for each individual question did not exceed 35% (Table 2).

**Table 2:** Knowledge about PrEP in the study population (*n* = 142)

		$\:n$	%
Pre-exposure prophylaxis (PrEP) for HIV consists of using antiretroviral (ARV) drugs to reduce the risk of acquiring HIV infection	True	46	32.4
	False	96	67.6
Only HIV-negative individuals can take PrEP	True	22	15.5
	False	120	84.5
Those at high risk for HIV are given priority access to PrEP	True	40	28.2
	False	102	71.8
Individuals on PrEP must take a pill at the same time every day	True	35	24.6
	False	107	75.4
Individuals on PrEP need to refill their pill supply regularly at a healthcare provider	True	43	30.3
	False	99	69.7
Individuals on PrEP must undergo regular testing for HIV and other sexually transmitted infections (STIs)	True	48	33.8
	False	94	66.2
Individuals on PrEP need to provide regular blood samples so that healthcare providers can monitor the drug’s effects on their organs	True	41	28.9
	False	101	71.1
Individuals on PrEP must receive regular counseling to help them adhere to their daily regimen	True	36	25.4
	False	106	74.6
Individuals on PrEP must receive regular counseling on how to reduce their risk of HIV infection through safer sex practices	True	45	31.7
	False	97	68.3
Pregnancy tests are always required for female users	True	23	16.2
	False	119	83.8
PrEP is not part of the combined HIV prevention strategies	True	40	28.2
	False	102	71.8
PrEP is highly effective when used correctly	True	48	33.8
	False	94	66.2
An HIV test result is required before starting PrEP	True	37	26.1
	False	105	73.9
The recommended PrEP regimen consists of a combination of the antiretroviral drugs tenofovir disoproxil fumarate (TDF) and emtricitabine (FTC) at a dosage of 300/200 mg	True	8	5.6
	False	134	94.4
For anal sex, seven consecutive days of PrEP use are required to reach maximum protective levels	True	16	11.3
	False	126	88.7
For vaginal sex, 20 consecutive days of PrEP use are required to reach maximum protective levels	True	15	10.6
	False	127	89.4
PrEP use is contraindicated during pregnancy	True	4	2.8
	False	138	97.2
PrEP should be prescribed with caution because users will abandon condom use, leading to an increase in other STIs, such as syphilis, gonorrhea, and chlamydia	True	12 130	8.5 91.5

*Abbreviations*: *HIV* Human immunodeficiency virus, *STI* Sexually transmitted infections, *PrEP* Pre-exposure prophylaxis

In the multivariable analysis, the following covariates were retained in the final logistic regression model for knowledge about PrEP: number of sexual partners in the last 6 months, sexual orientation, educational level, and perception of PrEP (Table 3). Only sexual orientation and the number of sexual partners were significantly associated with PrEP knowledge. The odds of having higher knowledge about PrEP were higher among LGBTQIA + participants (aOR: 4.06, 95% CI: 1.31–13.56) and among those who reported a greater number of sexual partners in the last 6 months (aOR: 1.52, 95% CI: 1.18–2.07). The Hosmer-Lemeshow (*p*-value = 0.976) and the le Cessie-van Houwelingen (p-value = 0.810) were non-significant, indicating a good model fit (AIC: 92.92)

 For the perception of PrEP, only age was retained in the final model and was significantly associated with the outcome (Table 3). The adjusted odds ratio indicates that younger participants were more likely to have a positive perception of PrEP. The Hosmer-Lemeshow (p-value = 0.426) and the le Cessie-van Houwelingen (p-value = 0.480) were non-significant, indicating a good model fit (AIC: 149.30).

**Table 3:** Logistic regression analysis of factors associated with high PrEP knowledge (≥ 60% accuracy) and a positive perception of PrEP

	**Knowledge**		**Perception**	
Covariates	*p*-value	aOR (95%CI)	*p*-value	aOR (95%CI)
Number of fixed sexual partnerships in the last 6 months	0.004	1.52 (1.18–2.07)	-	-
Sexual orientation (*LGBTQIA+*)	0.017	4.06 (1.31–13.56)	-	-
Educational level (*secondary education*)	0.156	0.22 (0.03–2.07)	-	-
Educational level (*higher education*)	0.844	0.83 (0.15–6.50)	-	-
Perception about PrEP (*positive*)	0.140	2.74 (0.77–11.91)	-	-
Age	-	-	0.021	0.96 (0.92–0.99)

*Abbreviations*: *aOR* Adjusted odds ratio, *PrEP* Pre-exposure prophylaxis, *LGBTQIA+* Lesbian, Gay, Bisexual, Transgender, Queer (or Questioning), Intersex, Asexual, and others (the plus sign represents additional identities not explicitly included in the acronym)

## Discussion

 The incorporation of PrEP within combined prevention strategies has positively impacted the control of HIV and other STI in several countries [7, 9, 18]. Innovations in PrEP use, including on-demand dosing [9], injectable formulations [19–22], and Descovy-based PrEP [23–26] (currently available only in the USA and associated with a lower risk of kidney toxicity), together with government initiatives to disseminate information, can directly influence users’ perception and reduce barriers to access and adherence, such as stigma [8, 11, 27, 28]. In this context, our study explored both individual and structural determinants of PrEP knowledge and perception among users of specialized STI and transgender healthcare services within Brazil’s public health system. Our findings revealed important gaps in knowledge despite the fact that most participants were eligible for PrEP use according to national guidelines. Younger participants and those identifying as men who have sex with men (MSM) showed a more favorable perception of PrEP, suggesting that age and sexual orientation play a relevant role in shaping awareness and acceptance. Conversely, limited information dissemination and the organizational structure of health services emerged as potential barriers to access and adherence. These findings reinforce the importance of strengthening counseling practices and expanding education strategies within routine healthcare, particularly in specialized services that serve populations at higher risk of HIV infection.

 The profile of participants in our study indicated that the vast majority were eligible to use PrEP according to the protocols in force at the time of data collection. However, none were currently using PrEP, as per the study's inclusion criteria. It is noteworthy that all individuals were approached in healthcare settings, which are appropriate venues for providing guidance on STI prevention measures. Institutionalized actions are crucial for imparting knowledge and updates on preventive measures during reception and counseling of individuals seeking health services. These efforts are essential for controlling and preventing HIV infection, particularly considering recent studies showing an increase in STI cases among young people, especially within the men who have sex with men (MSM) population [29, 31, 39].

 Although there is a federal law in Brazil that established the 'Red December' campaign – a national initiative for the prevention of HIV/AIDS and other sexually transmitted infections, which frequently mentions combined prevention strategies, including PrEP [32] – during the federal administration from 2019 to 2022, these campaigns and control strategy actions were heavily criticized. Specifically, there was no targeted promotion of PrEP, which was one of the innovative components of the combined prevention framework, unlike other well-known efforts, such as promoting condom use during Carnival. The Brazilian government's initiative in disseminating information about HIV/AIDS and its means of prevention, especially PrEP, through media most accessible to the general population remains limited, and the number of PrEP users in Brazil continues to be low, despite free access through the Unified Health System [33, 34]. In this sense, activities aimed at disseminating information to different age groups and contexts could contribute to greater adherence to combined prevention methods against STIs, especially considering that knowledge about this prophylaxis has been associated with a greater odds of PrEP use [34].

 Young and heterosexual individuals predominated in the study. The incidence of HIV has risen among cisgender men in the general population while decreasing among cisgender women in Brazil [35]. Although being a heterosexual man alone may not traditionally classify someone as part of a key population for priority STI prevention actions [36], the updated Brazilian protocol issued in 2022 recommends PrEP for all individuals at elevated risk of HIV infection [9]. This inclusive approach aims to effectively mitigate the spread of HIV across diverse demographics and risk profiles [9].

 Within this perspective of recommending PrEP for heterosexual people and considering the stigma that relates PrEP to key populations, which have historically been discriminated, the importance of welcoming and guidance on issues related to sexual health and HIV preventive measures and others STIs stands out in these groups. Stigma regarding the use of PrEP can lead to the possibility of risk compensation such as non-use or inadequate use of condoms and multiple sexual partners [37–39].

 Among respondents, 38% reported not using condoms during sexual intercourse. Individual and collective behaviors, such as inconsistent condom use, can be influenced by religious beliefs, dogmas, or doctrines, which may impact sexual and health practices and their consequences [40]. These factors should be considered when discussing our results, as nearly half of the participants identified as Catholic or Evangelical – religions with questionable positions regarding condom use. Consequently, the adoption of combined prevention methods, including PrEP, may also be hindered by stigma and discrimination related to gender and sexuality associated with religious beliefs. This can lead to resistance and a lack of preparedness in addressing the specific sexual health needs of different population groups [41–43].

 In marital and similar relationships, discussions about condom use are often absent, and the perceived vulnerability to STIs tends to decrease. Condom use in these contexts is frequently viewed primarily as a contraceptive method when other options are unavailable. Outside such contexts, its use may be interpreted as violating marital principles like respect and fidelity [44–46]. Consensual non-monogamous relationships also present unique challenges, as protection methods against STIs are often neglected, leading to decreased sexual self-control and increased exposure to STI risks [47–49].

 Positive perceptions of PrEP have notably increased among younger individuals, potentially influenced by social media platforms that disseminate information and facilitate the sharing of experiences related to HIV prevention [50], which corroborates our study. While stigma remains a barrier for vulnerable populations [51–53], advancements in access to information about this combined prevention method are evident. Educational initiatives using strategies such as posters, leaflets, campaigns, discussion groups, and social media promotions – especially those addressing sexual health – are crucial for raising awareness about STIs and their prevention.

 A large knowledge gap about PrEP among study individuals stands out. The willingness of healthcare professionals to promote and provide access to PrEP plays a crucial role. Studies highlight those contextual, organizational, and training factors influence PrEP recommendations. These findings underscore the importance of training programs that not only address clinical aspects but also tackle attitudes and values that may lead to reluctance in prescribing PrEP [54].

 Despite these efforts, significant barriers remain to the effective implementation of PrEP in health services, particularly for vulnerable groups. Stigma and discrimination related to gender identities and non-heteronormative sexualities, structural barriers, and the criminalization of sex work continue to hinder access to PrEP for these populations [55].

 Understanding the decision-making processes and the participation of stakeholders in PrEP implementation could inform future strategies for incorporating innovative HIV prevention tools for the most vulnerable groups [56]. Strengthening HIV prevention strategies, particularly among young people, is a priority. This includes increasing awareness among both the public and healthcare professionals and expanding access to innovative prevention options [57].

 The study's limitations were primarily associated with data collection during the COVID-19 pandemic, which potentially restricted users' access to services and information about PrEP. Social distancing measures also affected the scheduling of specific data collection periods in specialized services, potentially limiting the participation of certain population profiles. In this context of obtaining the data, it is worth considering that the results may have been impacted by the lack of dissemination of information on HIV/AIDS prevention to users, barriers to access to these services during the study period, profile of the population served or that accessed the health service, and professional commitment to address this topic with users. Other limitations include the small sample size and the recruitment of participants from only two specialized services with highly specific populations, which may limit the generalizability of the findings to other contexts. Also, we acknowledge that recruiting from referral services may introduce a selection bias, as the participants likely represent a population with a higher perception of risk and more engagement with the healthcare system compared to the general population eligible for PrEP. However, this strategy was the most feasible for accessing the target population, given that PrEP was exclusively available through the public health system in such specialized services at the time of data collection. To mitigate the potential impact of this bias on the internal validity of our findings and to control for confounding, we used multivariable logistic regression models to adjust for key variables such as age, sexual orientation, educational level, and sexual behavior. Despite these limitations, this sampling approach allowed for the characterization of the user profile within the actual clinical context where PrEP was offered, yielding relevant insights into the individual needs of these key groups and identifying major HIV/STI risk situations, thereby supporting targeted health interventions for the most vulnerable populations.

 Additionally, the threshold of 60% of correct answers adopted to classify adequate knowledge was based on a pragmatic criterion, given the lack of standardized cutoffs in the literature. This approach, although reasonable within this study’s context, may limit comparability with other populations or settings where different thresholds might yield distinct findings. Finally, the study was not powered a priori to detect small effect sizes. Although all consecutive eligible users who attended the services during the study period were included (*n* = 142), the relatively small sample may have reduced the power of the regression models and limited the precision of some estimates.

 This study offers insights into the epidemiological profile of current PrEP users, which may inform management strategies to improve service delivery and reduce barriers to prophylaxis access. The findings underscore the need for professional training that integrates technical knowledge, values and beliefs, and relevant public policies to adequately support individuals seeking care, particularly those from vulnerable populations. Furthermore, the importance of education and counseling for groups at highest risk of HIV and other STIs is highlighted, with the aim of expanding PrEP access and ultimately reducing infection incidence.

## Conclusions

 The predominant profile observed was that of young, heterosexual, non-white men with higher education, among whom condom use during sexual intercourse remained inconsistent. Limited knowledge about PrEP emerged as a major weakness, while younger users demonstrated a more favorable perception of the prophylaxis. These findings highlight the need for targeted health interventions that integrate combined STI prevention strategies, ensuring that PrEP is not linked to risk compensation or rising STI rates. Professional training is essential to better address the needs of this population, turning healthcare encounters into opportunities for health promotion and disease prevention. Continuous investment in education and health initiatives on PrEP is crucial to expand access and strengthen its coverage.

## Data Availability

All the data supporting the conclusions of this article are included in the manuscript.
